# Integration of Metabolic Modeling with Gene Co-expression Reveals Transcriptionally Programmed Reactions Explaining Robustness in *Mycobacterium tuberculosis*

**DOI:** 10.1038/srep23440

**Published:** 2016-03-22

**Authors:** Bhanwar Lal Puniya, Deepika Kulshreshtha, Inna Mittal, Ahmed Mobeen, Srinivasan Ramachandran

**Affiliations:** 1 CSIR-Institute of Genomics and Integrative Biology, Room No. 130, G.N. Ramachandran knowledge centre, Mathura Road, Near Sukhdev Vihar Bus Depot, New Delhi-110025, India

## Abstract

Robustness of metabolic networks is accomplished by gene regulation, modularity, re-routing of metabolites and plasticity. Here, we probed robustness against perturbations of biochemical reactions of *M. tuberculosis* in the form of predicting compensatory trends. In order to investigate the transcriptional programming of genes associated with correlated fluxes, we integrated with gene co-expression network. Knock down of the reactions NADH2r and ATPS responsible for producing the hub metabolites, and Central carbon metabolism had the highest proportion of their associated genes under transcriptional co-expression with genes of their flux correlated reactions. Reciprocal gene expression correlations were observed among compensatory routes, fresh activation of alternative routes and in the multi-copy genes of Cysteine synthase and of Phosphate transporter. Knock down of 46 reactions caused the activation of Isocitrate lyase or Malate synthase or both reactions, which are central to the persistent state of *M. tuberculosis*. A total of 30 new freshly activated routes including Cytochrome c oxidase, Lactate dehydrogenase, and Glycine cleavage system were predicted, which could be responsible for switching into dormant or persistent state. Thus, our integrated approach of exploring transcriptional programming of flux correlated reactions has the potential to unravel features of system architecture conferring robustness.

In the last decade, constraints based reconstruction and analysis (COBRA) approaches have been used to predict the behavior of several biological systems under a wide range of conditions. The COBRA toolbox and BiGG database are useful available resources of analysis methods and metabolic models^1^,^2^,^3^. Various applications of these models include wide range of studies encompassing metabolic engineering, model driven discoveries, cellular phenotype prediction, biological network analysis, evolution and interspecies interactions, drug target identification, and probing the robustness of metabolic networks^4^,^5^,^6^,^7^. Edwards and Palsson^7^ carried out robustness analysis of *E. coli* metabolic network and observed that the fluxes of transketolase and TCA cycle reactions could be reduced up to 15% and 19% respectively of the optimal value, without significantly altering growth^7^. These data show that in this ‘specific instance of design’ there exists a wide permissible range of values of metabolic flux for some biochemical reactions in the metabolic network, which results in near or total conservation of growth^8^.

Later, Reed and Palsson^9^ in a comprehensive analysis of the genome scale model of *E. coli* identified correlated reaction sets, which are reactions always used together and also referred to as fully coupled reactions^9^. They also showed that majority of the correlated reactions were in agreement with the regulatory architecture of the system. Rodrigues and Wagner^10^ showed that metabolic networks are endowed with great plasticity and networks of same phenotype could differ substantially in essential reactions and such differences encompass even important metabolic pathways such as central metabolism^10^,^11^. They suggested that this property could facilitate evolution of new metabolic functions and confer robustness against mutations.

As early as in 2001, Covert *et al.*, incorporated regulation of gene expression in FBA. They used conditions such as catabolic repression, diauxic shift and repression of amino acid biosynthesis^11^. In general, correlated reactions could be expected to be associated with gene expression correlation data. In yeast, a portion of the metabolic network, the central metabolism, showed high correlation between gene expression and correlated reaction sets^12^. Later Banta *et al.*^13^ and Schilling *et al.*^14^ observed that regulation of transcription may be important but not sufficient to regulate metabolic flux because a one-to-one correlation between the two variables could not be ascertained^13^,^14^. These observations suggest that the correlation between gene expression and metabolic flux could at best be expected to be moderate overall but subsections of the metabolic network may display high correlation. However, the general notion held is that integration of gene expression and metabolic flux data hold potential to offer insights into the dynamics of the biological systems responding to various stimuli^15^. In a recent study, in *Mycobacterium tuberculosis* we observed that the co-expression between genes associated with reactions having flux profile correlation – using gradual knockdown analysis - agreed most notably (greater than 50%) for highly connected hub genes in the genes co-expression network^16^.

The knocking down of a given reaction through constraining its flux in the genome scale metabolic network leads to altered fluxes of other reactions in the optimal solution. The altered fluxes of affected reactions can correlate either positively or negatively with the perturbed reaction. The negatively correlated reactions or fluxes of alternative reactions could compensate for the metabolic deficiency arising from perturbation. Therefore, examining the negatively correlated reactions offer a window to understand the robustness of a cellular system. In this work, we have identified all potentially negatively correlated reactions corresponding to gradual knock down of each of 386 reactions that carried non-zero flux in the wild type condition and also have gene-association rules. The correlated reaction pairs obtained from Flux Balance Analysis were re-examined using Flux Variability Analysis, and the reactions pairs that showed correlation in both the approaches were selected. We computed the co-expression status of genes associated with these correlated reactions from the gene expression data of *M. tuberculosis* exposed to various conditions and of *M. tuberculosis* gene knockout strain for hypoxia from publicly available 520 microarrays. Our goal was to examine the genetically pre-programmed architecture of the system offering potential to confer robustness on the metabolic system.

## Results

### Identifying flux correlation between reactions

**Figure 1:**
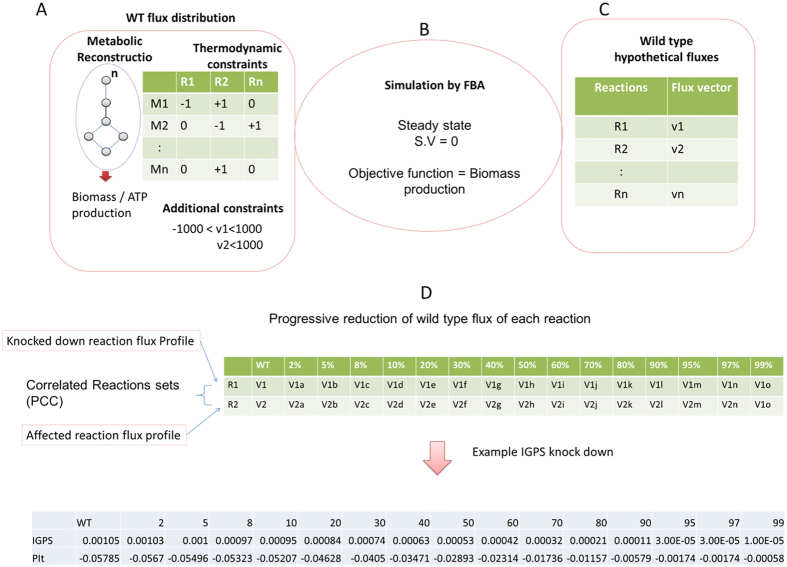
Identifying correlated reaction sets in Genome Scale Metabolic Reconstruction. (**A**,**B**) The genome scale metabolic network reconstruction updated *iNJ*661m (*iNJ*661mu) built using genome annotation, and bibliographic data. Using stoichiometric coefficient as thermodynamic constraint and other constraints as reaction bounds, these models could be used to simulate growth rate or biomass production (objective function in LP problem) *in silico* using Flux Balance Analysis approach assuming steady state. (**C**) Represents the wild type flux distribution of reactions. (**D**) Matrix of flux profiles of reactions shows flux profile of reaction R1 and flux profiles of affected reaction R2. All fluxes are shown V1 and V2 in WT and V1a is flux of R1 at 2% knock down and V2a is new flux of R2 at 2% flux reduction of WT flux of R1 and so on. Example is given as IGPS (Indole-3-glycerol phosphate synthase) knock down and affected reaction PIt (Inorganic phosphate transporter).

**Figure 2:**
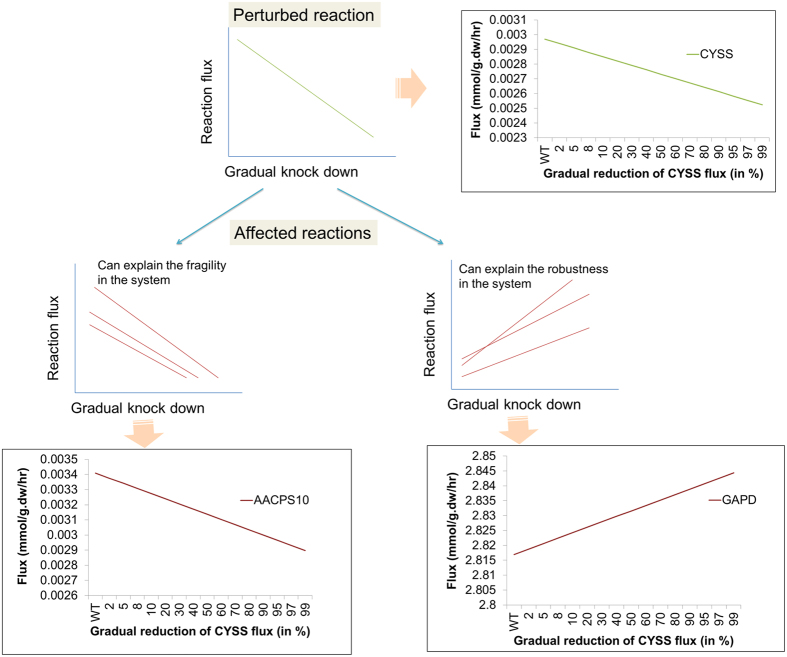
Illustrative example of positively and negatively correlated reactions in constraint based chemical reaction model. X – axis: Flux knockdown of flux of R1, and Y-axis: Flux values. In the upper panel the flux profile of knocked down reaction is shown. Lower panel shows two types of reactions. In positively correlated reactions (R2, R3 and R4) with reduced fluxes on knock down of R1, these constitute the fragility in the system. In negatively correlated reactions (R5, R6 and R7) with increased fluxes on knock down of reaction (R1), these constitute the robustness in the system. A real example of Cysteine synthase (CYSS) knock down and positively and negatively affected reactions is shown with the schematic representation.

Constraining flux of a reaction (knock down) mimics the inhibition of the activity of enzymes. An inherent feature of the metabolic pathways is the richness of junctions allowing for alternative routes^17^. Therefore, alteration in the flux of a given reaction can result in either decrement or increment of fluxes of other reactions. We knocked down 386 reactions progressively from 2–99% (see methods) of the original flux value in wild type condition and carried out flux balance analysis. In this approach we used the wild type flux of a reaction as a reference point and constrained the reaction by reducing to a value in the range 2–99%. We repeated the FBA simulation (Fig. 1, see methods for detail). By taking all perturbed points together (from 2% to 99%), we created two types of flux profiles (1) knocked down flux profile (2) affected reactions flux profiles. We computed Pearson’s correlation coefficient (PCC) between knocked down flux profile and the affected reactions flux profiles. The significant correlation between these show the coupling of fluxes when we perturb a particular reaction. A flux correlated reaction set is defined here as the set of reactions, whose fluxes are altered in response to the ‘knock down’ of the given reaction under the generated optimal solution for Biomass. The fluxes of altered reactions in response to the perturbation could decrease (positive) or increase (negative fluxes) (Fig. 2).

We selected reaction pairs (123834) with flux profiles having PCC with a minimum of ±0.5 and *P-value* < 0.05 (see Supplementary Fig. S1A). Of these, 386 reactions have gene-reaction association rules in the metabolic model. We considered knock down cases of these reactions with gene rules for integrating gene co-expression data. A total of 123,834 flux correlated reaction pairs were obtained by individually knocking down 386 internal reactions (Supplementary Table S1). Among these, 111,571 reaction pairs were positively correlated whereas 12,263 reaction pairs were negatively correlated considering absolute values of fluxes. It is evident that, an overwhelming proportion of reaction pairs (90.09%) were positively correlated and therefore they are associated with fragility of the system.

### Comparing reactions correlation using Flux Variability Analysis (FVA)

The correlated reaction pairs identified using the FBA could have the limitation as it uses only optimal solution. FVA calculates the maximal and minimal fluxes, which are consistent with maximal theoretical growth rate^18^. Thus to check the reactions’ correlations, we performed FVA at each point of perturbation using the same constraints (knock down from 2% to 99%). Then again we computed PCC between knocked down reaction’s FBA flux profile and flux profiles generated through FVA. FVA produces ranges of allowable fluxes (min and max). We generated two types of flux profiles each for minimum and for maximum flux points. The PCC cut off used was same as in the FBA analysis.

In the total of 111,571 positively correlated reactions’ pairs from FBA analysis, we observed 96943 (86.88%) reaction pairs showed positive correlations in FVA analysis also. On the other hand, in the cases of 12263 negatively correlated reaction pairs, we observed 8263 (67.38%) reactions’ pairs retained the negative correlation in FVA analysis. These numbers include the pairs in which knocked down flux profiles were significantly correlated either with the maximum allowable flux or with the minimum allowable flux in FVA analysis or with the both. These results showed the majority of correlated reactions pairs observed through FBA also agreed with flux variability analysis. The FVA results are shown in Supplementary Table S2. In the next sections, we used correlated reaction pairs, which were common in both FBA and FVA analysis (Supplementary Table S2).

### Pathways analysis of correlated reaction pairs

**Figure 3:**
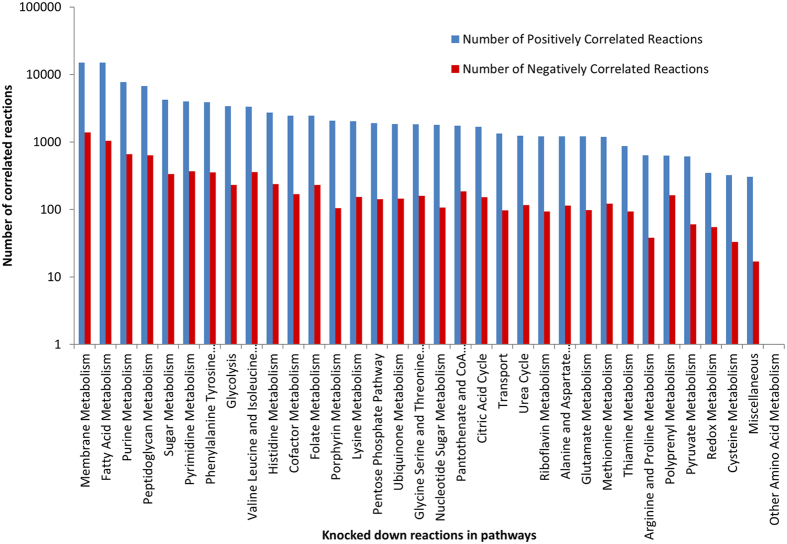
Numbers of reactions with correlated fluxes corresponding to knocked down of reactions in different metabolic pathways. X-axis: the pathways to which the knocked down reaction belongs and Y-axis: Numbers of reactions of affected pathways shown as bar histograms (Blue color: Positively correlated reactions and Red color: Negatively correlated reactions).

**Figure 4:**
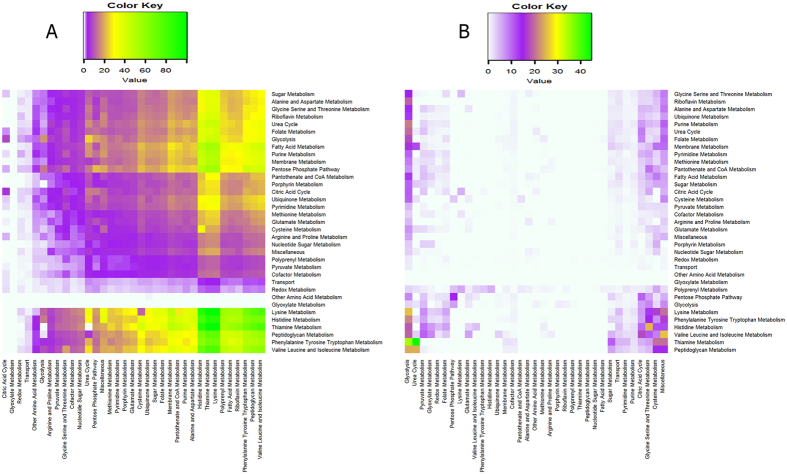
2-dimensional colourmap display of the scale of effects scored in terms of the numbers of reactions affected (reaction pairs) with respect to the total number of possible pairs in affected pathways on knockdown of reactions of a given pathway (in percentages) (**A**) positively correlated reactions, (**B**) negatively correlated reactions. The colour code corresponding to the numbers of affected reactions is shown in the corresponding scale bar. The knockdown pathways are in the rows and the affected pathways are in the columns.

The knocked down reactions were classified according to the pathways they belong to and the total number of reactions in the network correlating either positively or negatively are displayed in Fig. 3. The fold differences between positively and negatively flux correlated reactions vary in the range 1 to 19.70 with 78% of the cases falling within 10 fold difference. High numbers of correlated reaction pairs were in the following pathways: Membrane metabolism (15,075 positive and 1,391 negative pairs), Fatty Acid metabolism (15,006 positive and 1,044 negative pairs), and Purine metabolism (7,675 positive and 659 negative pairs) (Supplementary Table S3). The range of pathways spanned by the knock down of all reactions in a given pathway is displayed in Fig. 4A for positively correlated reactions and in Fig. 4B for negatively correlated reactions. The number of reactions affected in each pathway signifies the extent of the effect and percentage of reactions pairs formed relative to the total number of possible pairs in affected pathways are displayed in colour-coded format. In the case of positively correlated reactions, knockdowns of several pathways had wide ranging effects on many pathways some of them strikingly strong (Fig. 4A). Knockdowns of Fatty acid, Purine, Membrane, Pentose phosphate, Lysine, Histidine, Thiamine, Peptidoglycan and Valine Leucine and Isoleucine metabolic reactions generated affected reactions pairs in high proportions in some pathways most strikingly in Histidine, Thiamine and Lysine metabolic pathways. On the other extreme, the knockdowns of reactions of Glyoxylate, Other amino acid metabolic pathways did not affect other pathways. Conversely, the least affected pathways are Citric acid cycle, Glyoxylate, Redox and Transport (0–8.23% affected reaction pairs). The knockdowns of Fatty acid, Membrane, Purine and Pentose phosphate pathways have 28–56% pairs of total possible pairs. Similarly, knockdowns of Lysine, Histidine, Thiamine, Phenylalanine Tyrosine and Tryptophan, and Valine, Leucine and Isoleucine pathways had 37–100% of total pairs. Knockdowns in Lysine pathways have produced 100% affected reaction pairs in same pathway and Thiamine metabolism. Inversely, knockdowns in Thiamine metabolism has affected all the reactions in same pathway and Lysine metabolism. Knockdowns of reactions of Transport and Redox metabolism pathways had the lowest number of affected reactions pairs across all pathways, which range from 0–3.92%.

The negatively flux correlated reactions account for compensatory trends in the fluxes. Total number of reactions pairs formed vary from 0–45% of total possible pairs in affected pathways (Fig. 4B). The Glycolysis pathway was the most affected pathway among all the pathways in the system. The reactions of glycolytic pathway displays compensatory fluxes at high levels for the knock down of reactions of many metabolic pathways including Thiamine, Peptidoglycan, Lysine, Purine, Urea Cycle, Riboflavin , Folate, Fatty acid, Membrane. Conversely, knockdowns of reactions of Glycolysis result in marginal compensatory fluxes from Citric acid cycle, Glycine Serine Threonine, Pentose phosphate, Folate, Pyruvate metabolic pathways. The knockdowns of Glyoxylate and Other amino acid pathways had no compensation from any other pathway and the knockdowns of Redox metabolism and Transport were feebly compensated by Glycolysis. Knockdowns of Thiamine metabolism have most compensating reactions pairs in Urea cycle and Glycolysis 44.44% and 34.78% respectively and also from Cysteine and Miscellaneous pathways. Conversely, Thiamine metabolic pathway does not offer compensation to any other pathway knockdowns. Knockdowns of Valine Leucine and Isoleucine pathway formed 25.71% reactions pairs in Miscellaneous pathways. Knockdowns of Lysine metabolism, which formed 25.46% compensating reactions pairs in Glycolysis and knockdowns of Histidine pathway formed 25% reactions pairs in Glycine Serine and Threonine metabolism. Knockdowns of Peptidoglycan metabolism formed highest compensating pairs in Urea cycle and Glycolysis, which are 24.37% and 23.98% respectively. The remaining pathways in most instances (huge white area in the middle, Fig. 4B) either could not offer compensation or only feebly in some cases for the knockdowns of many pathways.

### Constructing the Gene co-expression network

After mean centered normalization, we selected expression profiles of 672 metabolic genes from 520 microarrays spanning a wide range of conditions of exposure of *M. tuberculosis* in order to include as much variability as afforded. The % Coefficient of variation (CV) ranges from 100 to 10^5^ along 672 genes across 520 samples (see Supplementary Fig. S1B). Embedded in this variability, co-expressed gene pairs with correlated expression can be considered informative as opposed to correlation between invariant or lowly varying expression profiles. Co-expressed genes within high variability of expression across many conditions are highly likely to be under co-regulation.

The gene co-expression network between 672 genes of metabolic network was constructed using the WGCNA package and we obtained two distinct modules of genes^19^. The network data first were cross examined with connections from Transcriptional Regulatory Network, STRING data or whether they had high topological weighted (0.06) co-expressed connections^20^,^21^. After applying these filters, we obtained 18,358 co-expressed connections between 651 genes (Supplementary Table S4). We present these results below.

### Co-expression between genes associated with same reaction (the case of multisubunit enzymes and duplicated genes)

In the *iNJ*661mu metabolic model, 193 reactions (125 unique gene rules) were associated with more than one gene. These reactions include NADH dehydrogenase (redox metabolism), ATP synthase (Purine metabolism), Mycobactin synthase (see Supplementary Table S5 for full list). 97 (49 unique gene rules) out of 193 reactions are associated with proteins from multiple genes functioning in multi-subunit enzymes (related exclusively by AND operators in the model). 63 reactions (55 unique gene rules) have genes related exclusively with OR operator signifying functional redundancy for each reaction. The remaining 33 out of 193 reactions had combinations of both AND and OR operators in the model. 312 out of 463 (68%) gene pairs among genes associated with same reactions showed co-expression in both modules taken together.

#### Multi-subunit enzymes

The genes associated with these reactions are found either in operons^22^ or in close genomic neighbourhood. A good majority of 80 out of 97 reactions (84%) catalyzed by multiple subunit enzymes showed positively correlated gene expression (Supplementary Table S5). Of these, 52 reactions (54.7%) had perfect positive co-expression (among all possible gene pairs) between genes associated with same reaction (topological overlap (TO) ranges from 0.006 to 0.32) and the rest 28 reactions had their gene pairs partially positively co-expressed (TO values ranges from 0.02 to 0.06). More than 50% of gene pairs showed co-expression in 12 of the partial cases. In the remaining 17 reactions out of 97, the correlation in expression between gene pairs was either not evident (11 reactions) or negative (6 reactions, TO values ranges from 0.008 to 0.06).

**Figure 5:**
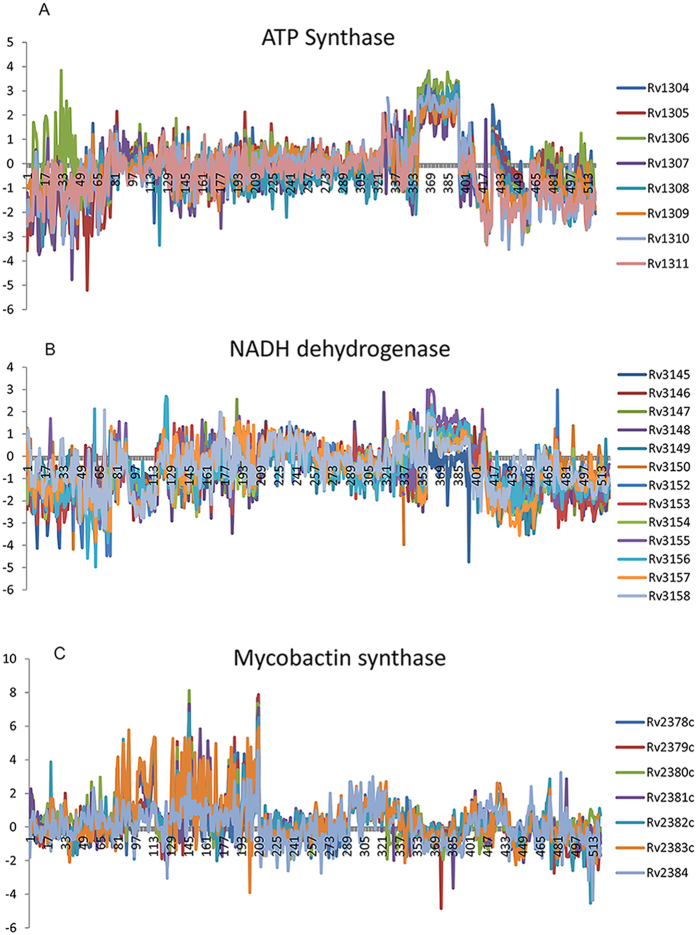
Gene expression patterns of subunits of enzymes (ATP Synthase, NADH dehydrogenase and Mycobactin synthase) encoded by multiple genes in 520 microarray samples. (X-axis = microarray data samples, and Y-axis = Z-score expression values) (**A**) The subunits of ATP synthase are encoded by 8 genes. All these 8 genes are co-expressed and have positive correlation in expression values. (**B**) The NADH dehydrogenase is encoded by 14 genes and all 14 genes are co-expressed and have positive correlation in expression values. (**C**) The Mycobactin synthase is encoded by 7 genes and all 7 genes are co-expressed and have positive correlation in expression values.

Some examples are noteworthy: ATP synthase (ATPS1) has multiple subunits encoded by 8 genes (*Rv1304, Rv1305*, *Rv1306*, *Rv1307*, *Rv1308*, *Rv1309*, *Rv1310*, *Rv1311*). All 28 pairs of these genes had positive correlation in the co-expression network (TO in the range 0.08 to 0.19). The Pearson’s correlation coefficients (PCC) ranged from 0.49 to 0.81 and were significant at p-value < 0.05. The Z-score expression values of all 8 genes of ATP synthase are shown in Fig. 5A and the correlation between their expression values is clearly evident. Another example is the NADH dehydrogenase (NADH5, NADH9, NADH10 and NADH2r), which is encoded by 14 genes (*Rv0082*, *Rv3145*, *Rv3146*, *Rv3147*, *Rv3148*, *Rv3149*, *Rv3150*, *Rv3152*, *Rv3153*, *Rv3154*, *Rv3155*, *Rv3156*, *Rv3157*, *Rv3158*). These genes form 91 pairs and 78 (85.71%) of these had positive correlations (TO in the range 0.015–0.21). The PCC values ranged from 0.47 to 0.85 and were significant at p-value < 0.05. The pattern of expression is displayed in Fig. 5B showing correlated expression. However, the gene *Rv0082* has lower negative correlation in expression (PCC range −0.12 to −0.24) with all other genes annotated as subunit of NADH dehydrogenase. The mycobactin synthase (MCBTS, MCBTS2, and MCBTS3) is encoded by the genes *Rv2378c, Rv2379c, Rv2380c, Rv2381c, Rv2382c, Rv2383c,* and *Rv2384*. All pairs between these genes showed positive correlation expression with PCC in the range of 0.54–0.84 and TO in the range 0.14–0.32. The patterns of expression of these genes are displayed in Fig. 5C showing correlated expression.

#### Isozymes

A total of 62 reactions are catalyzed separately by multiple single genes related by OR operator in the model. 16 (25%) of these showed co-expression among the associated gene pairs. We did not observe evidence of gene expression correlation between genes of remaining 46 reactions. Out of 16 reactions, 6 reactions had all gene pairs positively correlated expression and 2 reactions had negative correlation in expression between the respective single gene pairs. 8 reactions had their gene pairs partially correlated in expression. Of these 8 reactions, the reaction GTPCII (GTP cyclohydrolase II) and the reaction PDHc (pyruvate dehydrogenase dihydrolipoamide dehydrogenase) had negative correlation in expression between their genes. The reaction GTPCII is associated with two genes *Rv1415 or Rv1940,* (PCC − 0.18 and TO 0.01). The other reaction PDHc is associated with *Rv0462* or *Rv0843* (PCC − 0.35 and TO 0.06). Cysteine synthase is encoded individually by three genes (*Rv0848* OR *Rv1336* OR *Rv2334*). *Rv0848* is negatively correlated with *Rv2334* (PCC −0.49 and TO 0.09). On the other hand *Rv1336* and *Rv2334* were positively correlated in expression (PCC 0.29 and TO 0.04) and no significant correlation was observed between the genes *Rv0848* and *Rv1336*. Data for other reactions are given in Supplementary Table S5.

These results show that the genes coding for multi-subunit enzymes were significantly co-expressed (82% cases) and therefore are under significant transcriptional control. On the other hand multiple paralogous genes coding for enzymes with similar activity are co-expressed in a minority (25% cases) suggesting higher variability allowing economy through selected patterns of negatively correlated gene expression among the gene pairs.

### Integrating Co-expression between metabolic genes associated with flux correlated reactions

**Figure 6:**
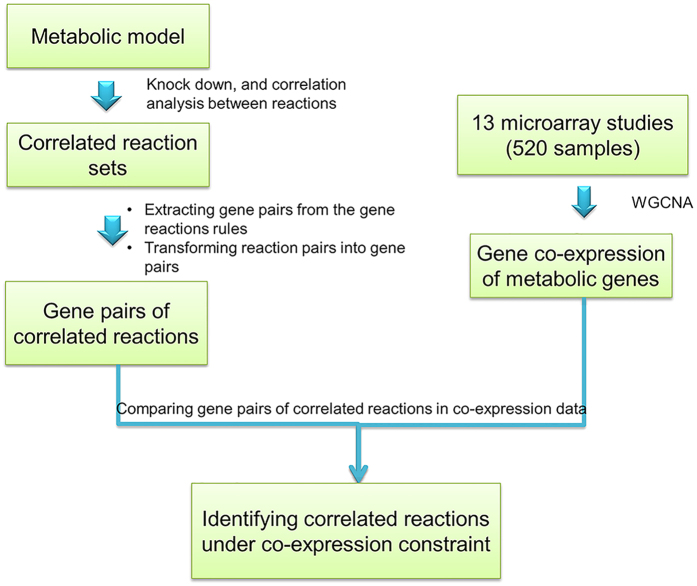
Work flow of integration of gene co-expression with reaction correlation.

Our goal was to investigate co-expression of genes associated with reactions with correlated fluxes in the reaction knock down analysis. We integrated flux correlated reactions with genes co-expression and examined the agreement between them. The workflow is presented in Fig. 6.

### Negatively flux correlated reactions had higher proportions of associated genes in co-expression

We examined the co-expressed connections between the gene pairs associated with the knocked down reaction and its negatively correlated reaction. In general, the proportion of reaction pairs in negatively correlated reactions (offering compensatory flux trends) under co-expression was 0.16 significantly higher than that of positively correlated reactions (0.11) (proportion test, two tailed p-value < 0.0001). These results show that the genes co-expression is more dominant in the negative flux correlated reactions. Because the negative flux correlated reactions are not directionally coupled, it might be possible that negative flux correlations require fine-tuned transcriptional regulation.

In the case of positively correlated reaction pairs, cysteine metabolism had the highest 43% of flux correlated reactions pairs under gene co-expression. Ratio of flux correlated reactions under co-expression for other pathways ranges from 4–22%. Out of knocked down of 386 reactions, 342 reactions had negatively correlated reactions. Of these, 269 negatively correlated reactions had at least one of their associated gene pairs co-expressed. 16 out of 269 reactions had shown gene co-expression for more than 50% of reactions (Table 1). 10 out of these 16 reactions had more than 10 reactions with increased fluxes on knock down. These reactions include ATP synthase, NADH dehydrogenase, Triose phosphate isomerase, Phosphoglycerate Kinase, Fumarase, Inorganic phosphate exchange diffusion, Glyceraldehyde-3-phosphate dehydrogenase, Phosphofructo kinase, and PPDIM synthesis (PPDIMAS and PPDIMAS) (Full list is given in Supplementary Table S6).

### Co-expressed flux correlated reactions varies across pathways

**Figure 7:**
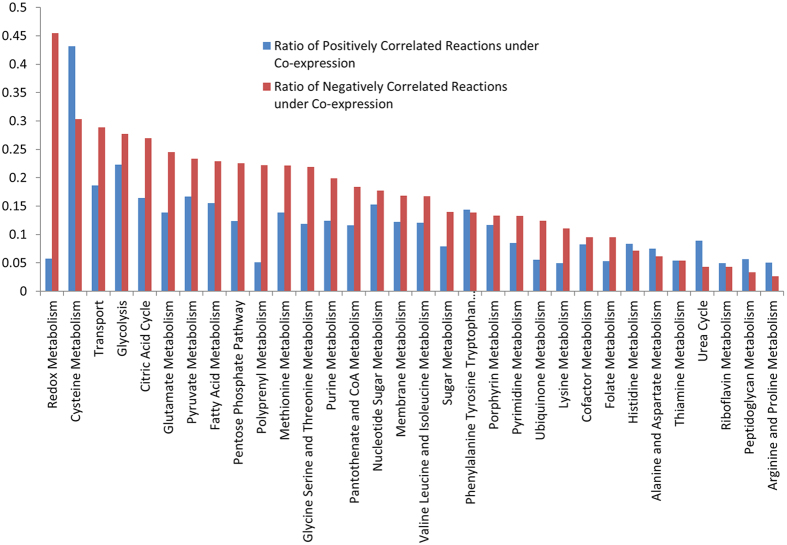
Proportion of reaction pairs under co-expression in metabolic pathways. X-axis: pathways and Y-axis: ratio of reaction pairs with associated genes co-expressed to the total number of reaction pairs. Blue colored bar: positively correlated reactions, Red colored bar: negatively correlated reactions.

The extent of genes co-expression is varied among pathways. When comparing the negatively correlated reactions with genes co-expression, it was evident that the redox metabolism topped the list of pathways at 45% of negatively correlated reactions found under genes co-expression. The cysteine metabolism, transport, glycolysis, and citric acid cycle are next top pathways with correlated reactions under gene co-expression ranged in 26–30%. The remaining 27 pathways had their 2–24% of negatively correlated reactions under genes co-expression (Fig. 7). In cases of the pathways Cysteine metabolism, Urea cycle, Peptidoglycan, Arginine and Proline, and Arginine and aspartate the positively correlated reactions showed higher proportion in genes co-expression. Histidine, Tryptophan, Tyrosine, and Phenylalanine metabolism, and thiamine metabolism had nearly equal positively correlated and negatively correlated reactions under genes co-expression (Fig. 7).

### Transcriptional programming of negatively flux correlated reactions

#### Reactions producing hub metabolites (ATP synthase and NADH dehydrogenase) had the most transcriptionally regulated negatively correlated fluxes

The knock down of ATP synthase (ATPS4r) reaction had 23 reactions with increased fluxes and 18 reactions (78.26%) had their associated genes co-expressed with *ATP synthase* genes. The knock down of NADH2r (NADH dehydrogenase) of redox metabolism had 29 reactions with increased fluxes (negatively correlated). The genes associated with 21 reactions of this set showed co-expression with the genes associated with NADH2r. In addition, the genes associated with 7 reactions were observed to be negatively co-expressed with the NADH2r genes.

The NADH dehydrogenase reaction builds electrochemical potential to produce ATP. The NADH and ATP are hub metabolites in the metabolic pathways because these metabolites participate in large number of reactions^23^.

#### Knock down of non-essential reactions had greater support from transcriptional co-expression

In 269 knocked down reactions with negatively correlated reactions sets, 220 were predicted to be essential in knock out simulation using FBA in the current model. We examined the proportion of genes associated with the negatively flux correlated reactions sets of knocked down of essential and non-essential reactions. The range of proportion of co-expressed genes associated with knocked down of essential reactions sets varied from 2.9% to 50% with median of 1.6%. Not a single essential reaction had more than 50% of their associated genes co-expressed in their flux correlated reactions sets. On the other hand the proportion of co-expressed genes associated with knocked down of non-essential reactions ranges from 3.3% to 100% with median of 27%. Negatively correlated reactions sets of 16 non-essential reactions showed at least 50% or above of their associated genes co-expressed. In addition to the reactions ATPS4r and NADH2r (described in previous section), non-essential reactions include central carbon metabolic reactions phosphoglycerate kinase (PGK), glyceraldehyde-3-phophate dehydrogenase (GAPD), fumarase (FUM), triosephosphate isomerase (TPI), phosphofuctokinase (PFK), and fumarate reductase (FRD), and phosphate transporter- inorganic phosphate transporter (PIt), and fatty acid metabolism (FAS161, FAS181, FACOAL181), which had high proportions of their associated genes co-expressed in their corresponding correlated reactions sets.

These data re-assert that in the knock down of essential reactions, the compensatory trends of flux through alternate metabolic pathways have feeble support of transcriptional co-expression, and likely contribute to fragility. On the other hand, the compensatory trends of flux through alternate reactions of knock down of non-essential reactions showed higher agreement with transcriptional co-expression of the corresponding associated genes and therefore likely contributes to the robustness in the metabolic system.

#### Negatively correlated reactions sets of Central carbon metabolism showed high transcriptional co-expression support

**Figure 8:**
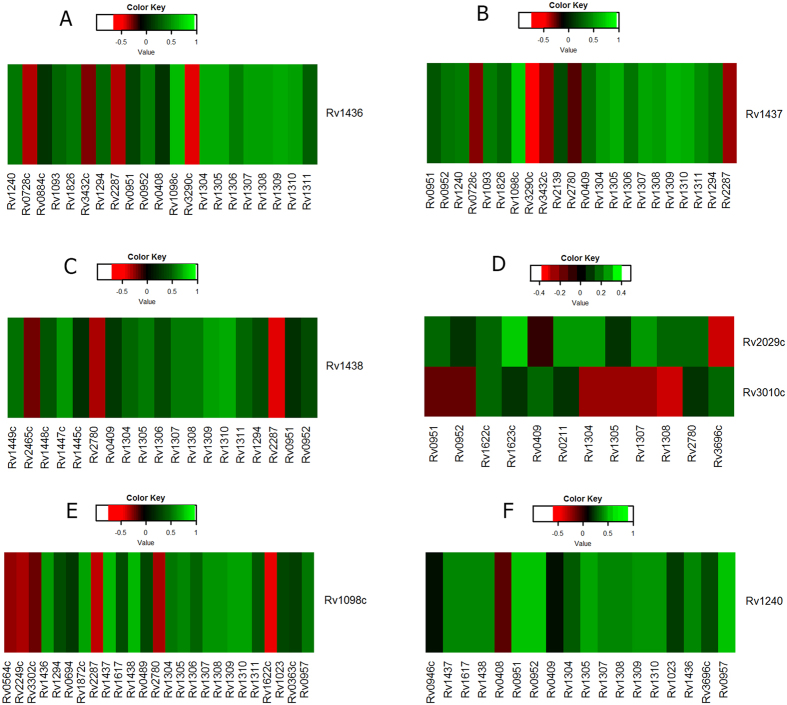
Gene expression correlation of genes associated with reactions of glycolysis and citric acid cycle pathways. The correlation values of one gene versus the rest are plotted in color coded format. The color code range bar corresponding to the correlation values is shown. (**A**) fumarase (**B**) malate dehydrogenase (**C**) glyceraldehyde-3-phosphate dehydrogenase (**D**) Phosphofructokinase (**E**) phosphoglycerate kinase (**F**) triose phosphate isomerase. Rows: genes of knocked down reaction, Columns: genes associated with negatively correlated flux of reactions with associated genes co-expressed. The scale bars are uniformly set −1 to +1 in colour scale ranging from red to green in all cases. However, in cases where the extreme values are absent, the colours are only assigned to the next minimum and maximum values available. Therefore the interpretations is that red signifies normalised low score values whereas green signifies normalised high score values.

Negatively correlated reactions sets of the reactions catalyzed by the enzymes triose phosphate isomerase (TPI), phosphoglycerate kinase (PGK), fumarase (FUM), glyceraldehyde-3-phosphate dehydrogenase (GAPD), Phosphofructokinase (PFK) and malate dehydrogenase (MDH) showed 66%, 63%, 61%, 59%, 53%, and 45% respectively of their associated genes under co-expression. The results are displayed in Fig. 8. Knock down of Fumarase reaction had 26 negatively correlated reactions, 16 of these had their associated genes co-expressed with the gene encoding Fumarase (Fig. 8A). The Malate dehydrogenase knock down had 26 negatively correlated reactions, of which 12 showed co-expression of their associated genes (Fig. 8B). The GAPD reaction knock down had 22 negatively correlated reactions, of which 13 had their associated genes co-expressed with the gene coding for GAPD (Fig. 8C). The PGK reaction knock down had 22 negatively correlated reactions, of which 14 showed their associated genes co-expressed with the gene coding for PGK (Fig. 8D). The TPI reaction knock down had 18 negatively correlated reactions, of which 12 showed their associated genes co-expressed with the gene coding for TPI (Fig. 8E). The PFK reaction knock down had 13 negatively correlated reactions, of which 7 showed their associated genes co-expressed with the gene coding for PFK (Fig. 8F).

It is noteworthy that FUM and MDH had common negatively correlated reactions with their associated genes co-expressed. These are AICART, ATPS4r, ENO, GAPD, PGK, PYK, and TPI. In the glycolytic pathway, the reactions GAPD, PGK, TPI, and PFK had their associated genes co-expressed in common with 2 reactions SUCOAS and ATPS4r. The GAPD, PGK, and TPI share 3 reactions SUCOAS, ATPS4r, and HSDy. GAPD and PGK had 10 common reactions (MDH, PGCD, GHMT2, GLYCL, GLUDC, HSDy, SUCOAS, FUM, ABTA, and ATPS4r). Interestingly, the citric acid cycle and glycolysis pathways commonly affect the ATPS4r, which is involved in energy metabolism.

#### Economic gene expression in PIt and CYSS reactions

##### Reciprocal co-regulation of two inorganic phosphate transporter genes Rv0545c and Rv2281

The Inorganic phosphate diffusion of phosphate from cytosol to extracellular is represented by the reversible reaction (Pi[cytosol] < − > Pi[extracellular]). This reaction is catalyzed by the products of two genes *Rv0545c* and *Rv2281*. In the wild type growth simulation, this reaction has negative flux value meaning that the phosphate ion transport flux is directed from extracellular to cytosol.

**Figure 9:**
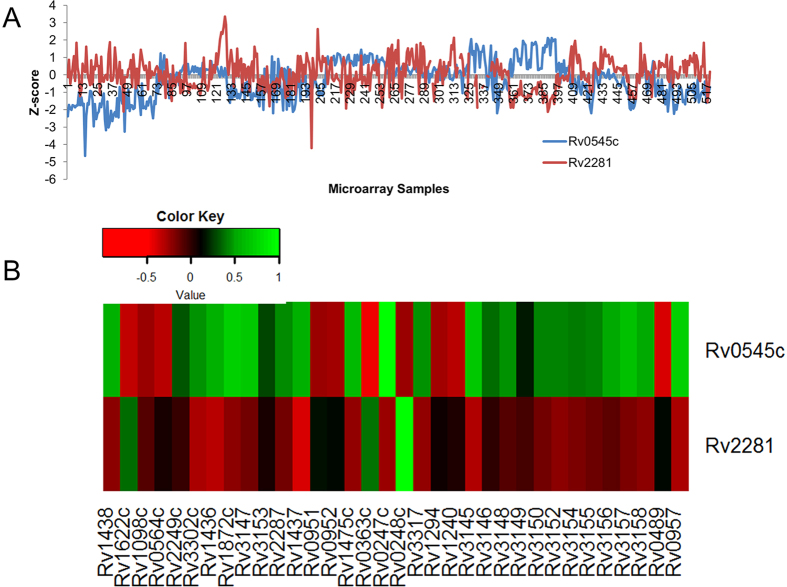
Reciprocal gene expression between the duplicated genes coding for inorganic phosphate transporter (PIt). (**A**) Patterns of gene expression between the two PIt genes Rv0545c and Rv2281 in 520 samples. X-axis: Microarray sample, Y-axis: Z-score of the gene expression values. (**B**) Heatmap of PCC between PIt associated genes and genes associated with negatively correlated flux reactions during Pit knockdown. Rows: *PIt* genes, Columns: genes associated with negatively correlated reactions.

The knock down of PIt increased fluxes of 28 reactions. Of these 28 reactions, 17 showed co-expression of associated genes with the genes coding for PIt. The results show that the PIt gene *Rv2281* showed negative PCC in gene expression (Fig. 9A) with 12 out of 41 co-expressed genes of negatively flux correlated reactions including TPI, CYTBD, FUM, G3PD1, GAPD, L_LACD2, NADH9, Nat3_1, and PGK. On the other hand, the gene *Rv0545c* showed positive PCC with the same genes catalyzing above reactions. However, *Rv0545c* showed negative PCC in gene expression with genes associated with three reactions including other transport reaction Nat3_1 (*Rv2287*), redox metabolism reaction CYTBD (*Rv1622c*), and glycerol-3-phosphate dehydrogenase NAD; G3PD1 (*Rv0564c* AND *Rv2249c* AND *R3302c*), in contrast the gene *Rv2281* showed positive PCC in gene expression with same genes (Fig. 9B). The results showed that the patterns of gene co-expression of *Rv0545c* and *Rv2281* with genes of all flux correlated reactions are reciprocally correlated. Also these two genes have negative PCC (PCC = −0.3) between their expression values. Therefore, it might be possible that these two genes function as separate transporters. Reciprocal coregulation of these genes with all other genes of flux correlated reactions with increased fluxes could account for robustness of this system under cellular economy.

##### Economy in gene expression in cysteine synthase system

The Cysteine synthase (CYSS) is encoded by each of the genes *Rv0848, Rv1336* and *Rv2334*. This reaction converts L-Serine into Cysteine. The knock down of cysteine synthase reaction had 24 reactions with elevated fluxes (negatively correlated). These negatively correlated reactions include 11 reactions of Glycolysis pathway, 5 reactions of Transport, and 1 reactions of Citric acid cycle. 10 (FRD5, GAPD, NAt3_1, TPI, LDH_L, PGK, ATPS4r, HSDy, SUCOAS, and PGM) of these reactions (41.66%) had their associated genes co-expressed with one of the cysteine synthase genes.

**Figure 10:**
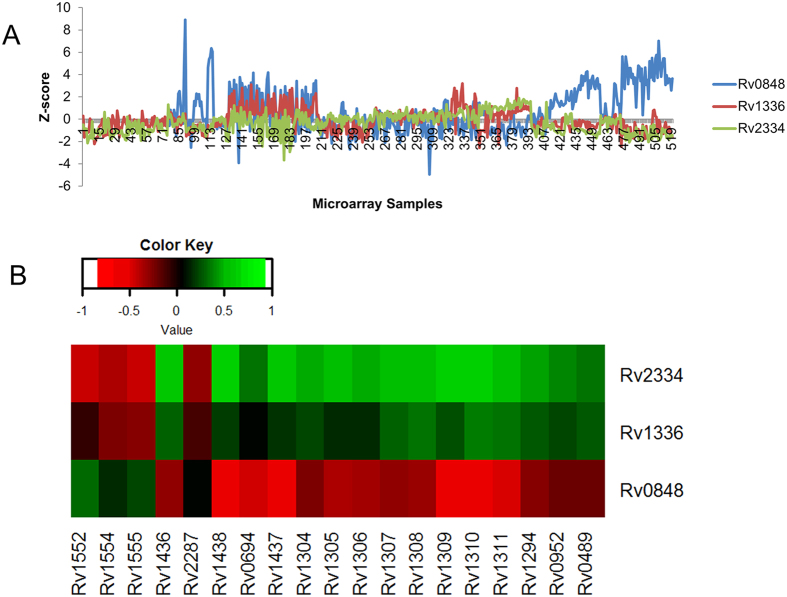
Reciprocal gene expression between the duplicated genes cysteine synthase (CYSS) (**A**) Gene expression patterns of three copies of cysteine synthase gene. The gene *Rv0848* is negatively correlated in expression with *Rv2334*. The genes *Rv2334* and *Rv1336* had positive correlation in expression. X-axis: Microarray sample, Y-axis: Z-score of the gene expression values. (**B**) Heatmap of PCC between CYSS associated genes and genes associated with negatively correlated flux reactions during CYSS knockdown. Rows: *CYSS* genes, Columns: genes associated with negatively correlated reactions.

The gene expression of *Rv0848* was negatively correlated with *Rv2334* whereas a positive correlation was observed between *Rv1336* and *Rv2334* (Fig. 10A). These two genes (*Rv1336* and *Rv2334*) also had similar gene expression correlations with all other genes of reactions with elevated fluxes (Fig. 10B). The expression of genes, which are positively correlated with both *Rv1336* and *Rv2334* include *Rv1436* (GAPD), *Rv1437* (PGK)*, Rv1438* (TPI), *Rv0489* (PGM), *Rv0952* (SUCOAS), *Rv0694* (LDH_L) and *Rv1304-Rv1311* (ATPS4r). The expression of these genes were negatively correlated with *Rv0848*. Conversely, expression of genes coding for fumarate reductase (*Rv1552* AND *Rv1554* AND *Rv1555*), and Nat3_1 (*Rv2287*) exhibited positive correlation with *Rv0848* but negative correlation with expression of the genes *Rv1336* and *Rv2334*.

The results suggest the co-expression of genes *Rv1336* and *Rv2334,* which negatively balances the expression of gene *Rv0848*. Reciprocal coregulation of these genes with all other genes of correlated reactions with increased fluxes could account for robustness of this system under cellular economy.

#### Activation of glyoxylate shunt enzymes iso-citrate lyase and malate synthase

In the knock down of reactions, we examined the fluxes of other reactions, which carried zero flux in wild type condition. These alternate fluxes represent activation of pathways in response to knock down. A total of 63 reactions which had zero flux in wild type activated against different reaction knock downs. We selected only those altered reactions, which showed associated genes co-expression with the knocked down reaction. A total of 32 freshly activated reactions with their associated genes co-expressed are shown in (Table 2). Five reactions were freshly activated with flux in response to knock down of greater than five reactions. These reactions include MALS (Malate Synthase), ICL (Isocitrate lyase), CYOb1, L_LACD3, and GLYCL. The reaction Malate synthase was activated in response to knock down of 34 reactions (the highest) whereas ICL was activated in response to knock down of 20 reactions. These results show that the fluxes through Malate synthase and Isocitrate lyase represent a common alternate route in response to knock down of many reactions in the metabolic network. ICL flux is common alternate to fatty acid metabolism (ACCC, ACChex, FACOAL160, MYCSacp50, MYCSacp56, MYCSacp58, PSD160), membrane metabolism (PREPTHS, PREPTHS2), Purine metabolism (ADSK, RNDR1), Valine Leucine and Isoleucine Metabolism (IPPMIa, IPPMIb), Tryptophan metabolism (IGPS), Nucleotide Sugar Metabolism (UDPG4E), Pantothenate and CoA Metabolism (PANTS), Pentose Phosphate Pathway (RPI), Pyrimidine Metabolism (TRDR) and Sugar Metabolism (UAGCVT). ICL and MALS have been shown to be responsible for persistent state of *M. tuberculosis* under low oxygen and nutrient limiting conditions^24^. ICL was shown to be responsible for the persistence state in mice by facilitating fatty acid metabolism, which is in agreement with our analysis^25^. MALS was activated against 34 knocked down reactions where it showed co-expression and showed negative gene co-expression with GLNS (glutamine synthetase). MALS and ICL forms glyoxylate shunt pathway, which serve as bypasses in TCA cycle. The activation of glyoxylate shunt pathway explains the tendency of *M. tuberculosis* to enter the persistence state when the metabolic pathways are perturbed through reaction knock downs.

In addition to MALS and ICL, we identified 30 more reactions, which were freshly activated against different reaction knock downs. These include CYO1b, L_LACD3, and GLYCL, which were activated against more than 10 knock down reactions. These 30 novel routes are likely associated with switching between normal and stress conditions (hypoxia, dormancy, persistence) of *M. tuberculosis*. The identified novel activated reactions along with MALS and ICL are displayed in Table 2. To investigate the association of freshly activated enzymes with stress conditions, we performed comprehensive literature search^26^. Knock down of gene *Ndk* (NDPK6) has shown significant reduction of persistence in *M. tuberculosis* in lungs of infected mice^27^. The activity of gene *gcvB* of glycine cleavage system was increased in non-replicating persistence^28^. The PNPs are listed among the top targets for *M. tuberculosis* persistence^29^. Sulfate adenylyl transferase genes (*Rv1285* and *Rv1286*) are part of stress induced operon^30^. Shi *et al.*, 2005 has shown that genes of cytochrome oxidase bd are required for adaptation to host immunity^31^. To further explore the experimental links of these enzymes, we queried gene expression data under stress conditions (i.e. hypoxia^32^, multiple stresses^33^, phosphate depletion^34^). Our results show that besides MALS and ICL, the genes of nine other reactions (CYO1b, GLYCL, ABTA, GCCa, L_LACt3, SADT2, CYTBD2, DHNPA, and DESAT16) were significantly up-regulated under hypoxia, multiple stresses, or phosphate depletion conditions (Table 2). Genes catalyzing ABTA (4-aminobutyrate transaminase) were up regulated in all the samples in GSE10391 (*in vitro* dormancy achieved by multiple stresses) and more than 1/3^rd^ samples of GSE9331 (Defined hypoxic model timecourse of H37Rv and H37Rv:deltadosR) (Table 2).

The observation that the activated reactions of the glyoxylate pathway and others had their associated genes under co-expression represents the inherent system architecture of the genes in the *M. tuberculosis* genome providing transcriptional co-expression support for the transition to persistent state under conditions of perturbation.

## Discussion

Unraveling the robustness of a system is key to understanding the pathogen and serve as potential for developing strategies to combat it. In this work we developed an integrated computational approach to the gene expression architectural feature along with flux correlations, which could explain the robustness in the cellular system of *M. tuberculosis*. These correlated reactions pairs on flux knock down were implemented to probe the dynamics of reactions’ fluxes. We used a direct way to test these features by carrying out knock down of the fluxes and identified the positively and negatively correlated reactions corresponding to each perturbed reaction. The negatively correlated reactions represent with elevated fluxes through alternate routes, which can be considered as compensatory trend in response to the deficiency arising by perturbation.

We used gene co-expression data to obtain agreement for flux correlated reactions. Various approaches have been developed to integrate gene expression data with metabolic models to build context specific metabolic model or applying regulatory information with metabolic model^35^,^36^. These approaches include Gene Inactivity Moderated by Metabolism and Expression (GIMME)^37^, *integrative metabolic analysis tool* (iMAT)^38^, Metabolic Adjustment by Differential Expression (MADE)^39^, E-flux^40^ and Probabilistic Regulation of Metabolism (PROM)^41^. The GIMME and iMAT use gene expression data to check functionality of reactions in metabolic network using expression level of genes associated with reactions. They create context specific metabolic models^35^,^36^. MADE requires two or more sets of microarray data to integrate differential expression with metabolic models. E-flux uses gene expression to constraint bounds of reactions which are lowly expressed. The probabilistic regulatory information inferred from gene expression data could be integrated using PROM method, which determines the probabilistic activity of a gene relative to activity of its transcription factor^36^. In the present work, we used co-regulatory information to test correlated reactions network of *M. tuberculosis.* Gene co-expression data can give information about relationship between genes functioning in different pathways. The co-expression between genes might be due to common regulation at transcriptional level or some other known genomic features such as genomic neighbourhood, phylogenetic co-occurrences or common pathways.

In the FBA simulation of knocked down phenotype, the objective function of cell (biomass production) is optimized. The FBA offers single optimal solution using linear programming. The predictions from FBA were already shown consistent with experimental data^42^. The approach of flux reduction was used for examining robustness of *E. coli*^7^. They showed that the system exhibited robustness against altered levels of fluxes of essential reactions. Although, the results of FBA have been shown to be consistent with experimental data, there is possibility of missing constraints in the metabolic model, which could affect solutions of FBA^43^. Further, we performed the FVA analysis and found the 3/4^th^ of the FBA generated correlated pairs had shown the agreement. To increase the confidence, we selected the correlated reaction pairs, which were common in both FBA and FVA approaches. Further, to extract meaningful information from flux balance analysis, we sought for agreement with transcriptional co-expression. As expected, we observed that greater than 82% of multi-subunit enzymes with neighboring and operonic genes showed positive co-expression. The proportion of associated genes co-expressed with correlated fluxes of reactions co-expression substantially varied across the pathways. In *E. coli*, Wessely *et al.*^44^ observed that pathways associated with high protein costs are controlled by fined tuned transcriptional mechanism^44^. Despite the partial agreement in whole system, we obtained good agreement in some pathways and reactions.

A noteworthy observation emerging from our work is that negatively flux correlated reactions had significantly high proportions of their associated genes in co-expression than positive flux correlated reactions. These results indicate that the system architecture of gene expression and regulation is aimed at controlling for the alternate routes of metabolism during perturbations. The proportion of positively correlated reactions having their associated genes under co-expression is not too low, quite acceptably, this arrangement suits the state during unperturbed state and when nutrients are available in abundance, it is all the more desirable that positively correlated reactions with flux have their associated genes co-expressed. It would therefore appear probable that a balance is to be achieved meeting requirements of good growth in luxurious conditions of nutrients and of reasonable growth to maintain survival under perturbations or other limiting conditions. We observed that reactions of some pathways e.g. ATP synthase of purine metabolism had 78% and NADH dehydrogenase of redox metabolism had 72% of their flux correlated reactions pairs had their associated genes co-expressed. These reactions form crucial parts of the metabolic network by providing the hub metabolites^23^. Therefore, the system appears to have invested a good amount of its architecture to keep these hub parts meeting the transcriptional co-regulation.

Another noteworthy observation emerging from our work is that the non-essential reactions showed higher proportions of associated genes co-expression in negatively correlated fluxes compared to essential reactions. It therefore appears that the non-essential reactions are associated with high degree of transcriptional co-expression and could confer robustness than the essential reactions.

The reciprocal regulation of gene expression is apparent providing for economy, alternate routes for fluxes and fresh activation of alternate routes of metabolism. Phosphate transporter and cysteine synthase had multiple paralogous genes with similar functional activities. A subset of the copies of the genes associated with reactions (CYSS and PIt) showed negative co-expression, which could explain economy in gene expression.

Most notably, several reactions had freshly activated flux during the knock down of reactions of many pathways including those of fatty acid metabolism and membrane metabolism. Activation of Isocitrate lyase and Malate synthase are among these, which are key enzymes implicated in the transition of *M. tuberculosis* to persistent state^25^,^45^. It was striking that the corresponding associated genes were under co-expression. We predicted 30 new freshly activated routes against perturbations. Literature survey and gene expression data analysis result shown that 12 out of these 30 enzymes were associated with non-replicating and/or stress conditions. These routes could be explored with the possibility of being molecular switch between normal state and persistent state. Thus it appears that the system architecture supports the transition to persistent state when metabolic networks are perturbed through knock down.

In conclusion, the genes associated with negatively flux correlated reactions (those offering compensatory fluxes) are co-expressed in higher proportions than the positively correlating reactions. The topmost genes co-expression agreement is among the genes associated with the reactions of hub metabolites. Reciprocal gene expression serves for economy among paralogous genes, for alternate routes of metabolic fluxes and for fresh activation of reactions associated with non-replicating persistent state. These system architectural features of the genes in the genome favours transition of *M. tuberculosis* to persistent state in the event of perturbation of metabolic networks. Thus our approach of integrating genes co-expression with flux balance analysis could reveal several insights into the system architecture.

## Methods

### Metabolic reconstruction of *M. tuberculosis*

Jamsidhi *et al.*^46^ and Beste *et al.*^47^ published genome scale metabolic reconstructions for *M. tuberculosis, iNJ661* and GSMN-TB respectively^46^,^47^. The *iNJ661* contains 661 genes and 939 reactions whereas GSMN-TB consists of 726 genes and 846 reactions. Later the *iNJ661* was further updated into *iNJ661m* considering bacterial growth in middlebrook 7H9 medium supplemented with glycerol and glucose and including reactions from GSMN-TB^48^. We further updated *iNJ661m* model by including novel reactions for genes with evidence in literature. Total 1208 genes were annotated for metabolism in TubercuList database^49^. A total of 472 genes are absent in all the models. A total of 183 genes are absent in *iNJ661m* but present in *GSMN-TB*. Out of these 183 genes 60 genes are present in KEGG database^50^. We used these 60 genes and identified reactions for these. We examined if metabolites of these reactions are connected in existing model. By analyzing all reactions, we observed that molybdenum co-factor biosynthesis is absent in *iNJ661m* model. These reactions were used to search literature for experimental evidences. The functional analysis of *moaD* gene showed presence of this pathway in *M. tuberculosis*^51^. We used these reactions from *GSMN-TB* to supplement in *iNJ661m* model. We slightly augmented the model by adding 8 reactions to the model. Out of these, one reaction is added as gap filling as the reaction of glyceraldehyde-3-phosphate to glycerol, which was absent in *iNJ661m* model. A total of 7 reactions for GTP to molybdenum cofactor synthesis were added including two reactions added for molybdate ion transport and exchange. The reaction of aldehyde dehydrogenase was added to fill the gap between glyceraldehyde-3-phosphate and glycerol in the metabolic model. This updated model *iNJ661mu* was used in this study (Supplementary Table S7). This model contains 1057 reactions and 672 genes and retaining the same biomass function. The model was simulated in Flux Balance Analysis and the biomass production rate was considered as objective function. The resultant model *iNJ661mu* was simulated in middlebrook 7H9 media using FBA approach. However, newly added reactions did not carry fluxes in wild type growth conditions when simulated using FBA. The newly added reaction showed flux when the network simulated using the FVA. This updation process was carried out to include new reactions in process towards completion of metabolic reconstruction. The growth rate produced by FBA simulation was the same growth (0.052 hr^−1^) as that of *iNJ*661 and *iNJ661m*.

### Identifying correlated reactions

We used *iNJ*661mu metabolic model to identify correlated reactions. To identify positively or negatively correlated reaction pairs metabolic reconstruction was first simulated using Flux Balance Analysis (FBA) to calculate wild type (WT; in optimal growth condition) flux distribution. This WT flux was used as reference starting point and reduced gradually. For example, for 2% knock down, we calculated 2% of WT flux and subtracted from the reference WT value. We used this equation (1) for calculated new flux of reaction.


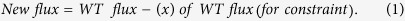


*x* = % reduction

e.g. For 2% knock down of a given reaction (2)


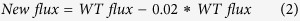


This new flux was used as new upper or lower (in case of reverse reaction, negative flux) bound for reaction. We created knocked down flux profile of reaction by reducing it 2%, 5%, 8%, 10%, 20%, 30%, 40%, 50%, 60%, 70%, 80%, 90%, 95%, 97% and 99% of its wild type flux. A flux profile is a vector, which has all new fluxes at each point of knock down. The fluxes for all other reactions were re-computed using the new bounds of the ‘knocked down’ reaction. Therefore, we simulated the model for 16 cycles for each reaction knock down using FBA approach.

All new fluxes of affected reactions were compiled in the form of a numerical matrix. The correlation between knocked down reaction flux profile and the affected reactions were computed using PCC. All significantly correlated reactions were used to generate reaction correlation matrix which contains PCC and P-value. All reactions, which had PCC > 0.5 were selected. The positive or negatively correlated reactions were computed using sign of PCC between knocked down reaction profile and affected reaction profile. Reactions with absolute PCC > 0.5 were selected.

Flux variability analysis was performed using the same approach. The gradual knock down of wild type flux values was done and used as the constraints at each point. After each simulation, we collected maximum and minimum allowable fluxes. To identify correlated fluxes, we created two separate flux profiles max and computed PCC with knocked down flux profiles. The reactions which showed the significant PCC either with maximum flux profile or with minimum flux profile or with the both, were collected. These correlated pairs then compared with correlated pairs generated using FBA. We selected reaction pairs, which showed correlation in both FBA and FVA approaches.

### Gene co-expression network construction

#### Selecting microarray data and preprocessing

A total of 113 GSE series were present in the GEO database (as on January 17, 2013) on *M. tuberculosis*^52^. Of these, 78 GSE series have normalized log ratios for experimental cases and normal controls. We selected these 78 GSE series and selected 13 experiments, with exposure of *M. tuberculosis* with different environmental conditions. We considered environmental conditions (different growth phases, stresses, dormancy, and in response to host immunity) as source of variability in data. We excluded all experiments treated with drugs, overexpression, and specific gene knock outs without combination of different environmental conditions. We considered two knock out studies, which were carried out with combination of other environmental conditions. In GSE9331 dosR mutants were studied with hypoxia in which hypoxic response was captured in normal and dosR mutants at different time points^32^. We included data on log phase growth, Stationary phase, hypoxic conditions with or without dosR, dormancy by multiple stresses, NO treatment, dosR/dosS mutants with CO treatment, inside macrophage, phosphate depletion, response to lung surfactants^32^,^33^,^34^,^53^,^54^,^55^,^56^,^57^,^58^,^59^,^60^. All data were normalized ratios with background corrections between cases vs control. The sample and experimental information of these experiments is summarized in Table 3.

All 521 microarray samples present in 13 GSE series were preprocessed. Replicated probes were averaged in GSE10391, GSE11096 and GSE14840. The unique probe IDs were mapped to Rv IDs in all experiments and combined all the GSE series. Cross study normalization was carried out using Z-score transformation^61^. The Z-score for each gene was computed by subtracting the sample mean from current values and then dividing it by the standard deviation of all values of expression of all genes in a sample. This method normalizes all samples to zero mean and standard deviation of one. The boxplot of all samples is displayed in Supplementary Fig. S1C. After cross study normalization, we selected all metabolic genes which are present in the metabolic reconstruction *iNJ*661mu. We combined all experiments for 672 metabolic genes to create a single matrix.

#### Inferring Co-expression between metabolic genes

The co-expression between 672 metabolic genes was computed using Pearson’s Correlation Coefficient (PCC). One sample (GSM538999 of GSE21590) was removed due to the large number of missing values. Because we had large sample size of 520 microarrays with high biological variability estimation of correlation are likely to be informative between genes.

The Weighted Gene Co-expression Network Analysis (WGCNA) method was used for co-expression network construction^19^. The WGCNA uses topological overlap (TO) as measure of similarity between genes, which is robust measure than correlation^19^. The TO measure uses correlations between genes as well as their directly connected neighbours. The weighted gene co-expression network for all 672 metabolic genes was constructed. This network is unsigned. The soft thresholding power at which the network was fitted to scale free topology at approximately (R^2^ > 0.85) was 3. R^2^ is linear regression model fitting index. It quantifies extent of fitting to scale free topology (see Supplementary Fig. S1D). We obtained two modules but we considered all topological overlap between genes because we constructed co-expression network using metabolic genes only.

To further check the validity of co-expression network we compared this network with other data sources including Transcriptional Regulatory (TR) Network of *M. tuberculosis* and protein association data from STRING database^20^,^21^. The TR network was constructed using multiple experimental data and has information on genes under similar transcriptional regulation. The STRING database predicts protein association on the basis of conserved genomic neighbourhood, gene-gene co-occurrences, conserved co-expression, homology and literature based information^20^. We used high confidence connections from STRING database (confidence score >0.8). The distribution of common connections at different topological overlap (TO) weight cut-off with TR and STRING data sources is shown in Supplementary Fig. S1E. The ratio of common connections to total co-expressed connections was increased with high TO. All connections with TO cut-off 0.06 had significant PCCs between all the gene pairs (see Supplementary Fig. S1F). We considered all significant correlations with P-value < 0.05 (TO minimum of 0.06). The absolute PCC at this minimum value of TO range 0.09 to 0.91. Additionally, we enriched connections with lower TO but were supported with STRING database at confidence score cutoff 0.8 and TR network^20^,^21^. The approach of integration of gene co-expression with other data sources was similar to the approach used by Puniya *et al.*^16^. Therefore, the final network contains all high weighted (minimum TO 0.06) connections and low TO but supported connections^16^.

#### Validation of co-expression network using random sampling

We also carried out random sampling of co-expressed connections (1000 times) to check the chance effect. The equal numbers of connections of final network were randomly sampled between same genes 1000 times. These random connections were compared with STRING database. The common connections with randomly sampled connections and STRING database were noted. The common connections of original network with STRING, and random network with STRING were compared. The mean of 1000 random sampling were considered and significance was tested if the original score was greater than mean + 3SD of the randomly sampled cases.

#### Validation of co-expression network with multi-subunit enzymes (Positive Dataset)

The genes in metabolic reaction networks are represented as gene-protein-reaction (GPR) association rules. A total of 730 reactions in metabolic network *iNJ*661mu have associated gene rules of which 534 reactions have single gene associated rules. The remaining 197 reactions have gene rules (GPR associations) in the form of Boolean logics. First we checked gene expression correlation between genes associated in Boolean rules of same reactions. We used this as positive dataset, to check the correlation between genes associated with same reactions. The genes of same reactions were searched in co-expression data. To check significance of results we also compared gene co-expression between ‘AND’ Boolean logics (multi-subunit enzymes) with ‘OR’ operator Boolean rules. The PCCs were computed for co-expressed gene pairs and all significant correlations were considered (P-value < 0.05).

### Integration of gene co-expression with reaction flux correlation

Two correlated reactions were transformed into gene pairs by combining associated genes of correlated reactions. Therefore, these gene pairs include one gene from each reaction in the correlated reaction pair. In general, the numbers of gene pairs are greater than reaction pairs because some reactions are associated with multiple genes. In multiple gene reactions, reactions with significant co-expression in at least one pair of genes were selected. All genes of AND, OR Boolean logic was compared by forming pairs among genes of correlated reactions in flux.

The reactions that had at least one pair of genes co-expressed were considered. The total numbers of reactions pairs with co-expressed genes were calculated and proportions were computed from all correlated reaction pairs formed in knock down experiments. To check the amount of correlated reaction pairs under co-expression constraint, we compared positively and negatively correlated reactions separately. First, we checked these reactions for unsigned co-expression between genes. The differences of proportions under co-expression constraint were computed using *prop.test* function in R^62^. For significant differences the P-value < 0.05 were considered. The proportion of reaction pairs, which showed co-expression was calculated using background of total number of pairs.

To check the degree of co-expression of correlated reactions across pathways, we mapped knocked down reactions with pathways and counted total numbers of altered reactions in different pathways. The negatively correlated reactions were extracted for further analysis. Essentiality of reactions is predicted by knockout using FBA simulation. This was carried out by setting up reaction’s bounds to zero. Reaction was considered as essential if simulation after knockout cannot produce biomass. Rest of the reactions considered as non-essential. Validity of knockout simulation using these models of *M. tuberculosis* was studied in previous works and found to be in agreement with large scale gene deletion data through transposons *in situ* hybridization data (TraSH)^63^.

Metabolic model analysis and simulation was performed using COBRA toolbox V2^2^,^3^,^64^ in MATLAB 10.0. Gurobi5 solver was used in FBA simulation^65^. Other statistical analyses were performed in R^62^ and some data mapping were done in Microsoft Excel 2010.

## Additional Information

**How to cite this article**: Puniya, B. L. *et al.* Integration of Metabolic Modeling with Gene Co-expression Reveals Transcriptionally Programmed Reactions Explaining Robustness in *Mycobacterium tuberculosis. Sci. Rep.* **6**, 23440; doi: 10.1038/srep23440 (2016).

## Supplementary Material

Supplementary Information

Supplementary Table S1

Supplementary Table S2

Supplementary Table S3

Supplementary Table S4

Supplementary Table S5

Supplementary Table S6

Supplementary Table S7

## Figures and Tables

**Table 1:** Top transcriptionally regulated negatively correlated reactions showing gene co-expression with at least 50% of total reactions.

Knocked down reaction	No. of negatively correlated reactions	No. of reactions showed gene co-expression with knocked down reaction	Reactions with associated genes with Negatively correlated (PCC) expression
**ATPS4r** (ATP synthase four protons for one ATP)	23	18	8
**NADH2r** (NADH dehydrogenase)	29	21	7
**TPI** (Triose phosphate isomerase)	18	12	3
**PGK** (Phosphoglycerate kinase)	22	14	5
**FUM** (Fumarase)	26	16	4
**PIt** (Inorganic phosphate exchange diffusion)	28	17	9
**GAPD** (Glyceraldehyde-3-phosphate dehydrogenase)	22	13	4
**PFK** (Phosphofructokinase)	13	7	0
**PDIMAS** (PDIM A synthesis *Mycobacterium tuberculosis*)	22	11	2
**PPDIMAS** (PPDIM synthesis *Mycobacterium tuberculosis*)	22	11	2
**FAS161** (Fatty acid synthase n C161)	4	4	1
**FAS181** (Fatty acid synthase n C181)	4	4	1
**FACOAL181** (Fatty acid coa ligase octadecenoate)	3	2	0
**ADK4** (Adentylate kinase ITP)	1	1	0
**MTHFD** (Methylenetetrahydrofolate dehydrogenase NADP)	1	1	0
**FRD** (Fumarate reductase)	2	1	1

**Table 2:** Newly activated reactions in response to reaction knock down.

Activated Enzymes	Against *in silico* knocked down of reaction(s)*	Remark (Literature Survey)	Gene expression data analysis (Genes up-regulated in stress conditions)
**MALS** (Malate Synthase) (*Rv1837c*)	Cofactor Metabolism (AHMMPS, GLUTRR), Fatty acid metabolism (ACCC, ACChex, FAMPL1, FAMPL2, FAMPL3, FAMPL4, FAMPL5, FAS100, FAS120, FAS140, FAS200, FAS240_L, FAS80_L, MYC1CYC1, MYCON1, MYCSacp50, MYCSacp56, MYCSacp58), Glutamate Metabolism (**GLNS**), Histidine Metabolism (IGPDH), Lysine Metabolism (DHDPS), Membrane metabolism (PREPTHS, PREPTHS2), Methionine Metabolism (AHC), Other Amino Acid Metabolism(PAPR), Pantothenate and CoA Metabolism (MOHMT), Phenylalanine Tyrosine Tryptophan Metabolism (ANS, IGPS), Porphyrin Metabolism (UPP3S), Valine Leucine and Isoleucine Metabolism (ACLS, IPPS)	Reported to active against persistent state of *M.tuberculosis*^24^	
**CYO1b** (Cytochrome c oxidase) (*Rv1451 and Rv2193 and Rv2200c and Rv3043c*)	Cofactor Metabolism (GLUTRR, THMDP, UDCPDP), Fatty Acid Metabolism (FAMPL1, FAMPL2, FAMPL3, FAMPL4, FAMPL5, FAS240_L, FAS260, MYC1CYC1, **MYC2CYC1**, MYCON1, MYCON3, MYCON4, MYCON5, MYCSacp50, MYCSacp56, MYCSacp58, **PSD160**), Membrane Metabolism (TRE6PS), Phenylalanine Tyrosine Tryptophan Metabolism (CHORM, DDPA), Polyprenyl Metabolism (DCPDP), Porphyrin Metabolism (G1SATi, UPP3S, UPPDC1, HMBS), Sugar Metabolism (PMANM, UAGDP), Valine Leucine and Isoleucine Metabolism (KARA2i)		***Phosphate depletion *****(*****GSE14840*****):** Up-regulated in 3 samples (Out of 6 samples).
**ICL** (iso-citrate lyase) (*Rv1915* and *Rv1916*) or (*Rv0467*)	Cofactor Metabolism (DMATT), Fatty Acid Metabolism (**ACCC, ACChex, MYCSacp50, MYCSacp56, MYCSacp58, PSD160,** FACOAL160), Membrane Metabolism **(PREPTHS, PREPTHS2),** Nucleotide Sugar Metabolism (UDPG4E), Pantothenate and CoA Metabolism (PANTS), Pentose Phosphate Pathway (RPI), Phenylalanine Tyrosine Tryptophan Metabolism (IGPS), Purine Metabolism (ADSK, RNDR1), Pyrimidine Metabolism (TRDR), Sugar Metabolism (UAGCVT), Valine Leucine and Isoleucine Metabolism (IPPMIa, IPPMIb)	Reported to active against persistent state of *M. tuberculosis*^24^; Activation facilitates fatty acid metabolism^25^	
**L_LACD3** (Lactate dehydrogenase) (*Rv1872c*)	Fatty Acid Metabolism (FACOAL160, FAS100, FAS120, FAS140, FAS200, FAS80_L), Glutamate Metabolism (GLUDx), Lysine Metabolism (DHDPS), Membrane Metabolism (PREPTHS, PREPTHS2), Methionine Metabolism (AHC), Pantothenate and CoA Metabolism (MOHMT, PNTK, PPCDC), Phenylalanine Tyrosine Tryptophan Metabolism (ANS, IGPS), Purine Metabolism (**ADSK**, ATPS4r)		
**GLYCL** (Glycine cleavage system) (*Rv1826*)	Fatty Acid Metabolism (FACOAL160, FAS100, FAS120, FAS140, FAS200, FAS80_L), Glycolysis (GAPD, PGK), Pantothenate and CoA Metabolism (PANTS), Phenylalanine Tyrosine Tryptophan Metabolism (CHORM, IGPS), Purine Metabolism **(RNDR1),** Pyrimidine Metabolism (NDPK2, NDPK4, **TRDR),** Transport (**NH4t)**, Valine Leucine and Isoleucine Metabolism **(DHAD1, **KARA1i)		***Hypoxia*** **(*****GSE9331*****):** Up-regulated in 15 samples (Out of 50 samples).
**ABTA** (4-aminobutyrate transaminase) (*Rv2589* or *Rv3290*)	Glycolysis **(GAPD, PGK),** Transport (NH4t), Valine Leucine and Isoleucine Metabolism(**IPMD**, IPPMIa, IPPMIb, IPPS)		***Multiple stresses*(*GSE10391*):** Up-regulated in 28 samples (out of 28 samples).***Hypoxia*** **(*****GSE9331*****):** Up-regulated in 39 samples (out if 50 samples) ***Phosphate depletion*****(*****GSE14840*****):** Up-regulated in 3 samples (out of 6 samples)
**G6PDH2** (glucose-6-phosphate dehydrogenase) (*Rv1447c* or *Rv1121*)	Glycolysis (PGI, TPI), Pentose Phosphate Pathway (RPE, TALA, TKT1, TKT2)		
**PGL** (6-phosphogluconolactonase) (*Rv1445c*)	Glycolysis (**PGI,**TPI), Pentose Phosphate Pathway (TKT1, TKT2)		
**GLUDC** (Glutamate decarboxylase) (*Rv3432c*)	Alanine and Aspartate Metabolism (ASNS1), Glycolysis (**GAPD, PGK**)		
**GND** (phosphogluconate dehydrogenase) (*Rv1844c* or *Rv1122*)	Pentose Phosphate Pathway (RPE, TKT1, TKT2)		
**NDPK6** (nucleoside di-phosphate kinase ATPdUDP) (*Rv2445c*)	Pantothenate and CoA Metabolism (MOHMT), Purine Metabolis (PANTS)	Knock down of Ndk significantly reduced *M. tuberculosis* persistence in lungs of infected mouse^27^	
**GCCb** (glycine cleavage complex) (*Rv2211c*)	Phenylalanine Tyrosine Tryptophan Metabolism (ANPRT), Valine Leucine and Isoleucine Metabolism (ACLS)	Activity of gcvB increased in non-replicating persistence^28^.	
L_**LACD** (L-Lactate dehydrogenase irreversible) (*Rv0694*)	Membrane Metabolism (TRE6PS), Phenylalanine Tyrosine Tryptophan Metabolism (CHORM)		
**HPPK** (2-amino-4-hydroxy-6-hydroxymethyldihydropteridine di-phosphokinase) (*Rv3606c*)	Folate Metabolism (DHNPA2, DHPS2)		
**DHORD2** (dihydoorotic acid dehydrogenase quinone8) (*Rv2139*)	Glycolysis(PGK), Histidine Metabolism (IG3PS)		
**DESAT18** (stearoyl-CoA desaturase) (*Rv1094* or *Rv0824c*)	Fatty Acid Metabolism (FACOAL181, FAS181)		
**GCCa** (glycine cleavage complex) (*Rv1832*)	Pyrimidine Metabolism (**DHORTS**)		***Hypoxia*****(*****GSE9331*****):** Up-regulated in 21 samples (out of 50 samples).
**P5CR** (pyrroline-5-carboxylate reductase) (*Rv0500*)	Valine Leucine and Isoleucine Metabolism (KARA1i)		
**L_LACt3** (L-lactate transport) (*Rv0191*)	Purine Metabolism (**ATPS4r**)		***Hypoxia*****(*****GSE9331*****):** Up-regulated in 17 samples (out of 50 samples).
**PUNP7** (purine nucleoside phosphorylase Xanthosine) (*Rv3307*)	Purine Metabolism (**ADPT)**	PNP are listed among top targets for *M. tuberculosis* persistence^29^	
**SADT2** (Sulfate adenyltransferase) (*Rv1285* and *Rv1286*)	Purine Metabolism (SADT)	CysD and CysN both are part of stress induced operon^30^.	***Multiple stresses*****(*****GSE10391*****):** Up-regulated in 7 samples (out of 28 samples). ***Hypoxia*** **(*****GSE9331*****):** Up-regulated in 46 and 42 samples (out of 50 samples).
**FOLD3** (dihydropteroate synthase) (*Rv1207* or *Rv3608c*)	Folate Metabolism (DHNPA2)		
**G5SD2** (glutamate-5-semialdehyde dehydrogenase) (*Rv2427c*)	Valine Leucine and Isoleucine Metabolism (KARA1i)		
**CYTBD2** (cytochrome oxidase bd menaquinol 8 2 protons) (*Rv1620c* and *Rv1621c* and *Rv1622c* and *Rv1623c*)	Glycolysis (PFK)	Required for *M. tuberculosis* adaptation to host immunity^31^.	***Hypoxia*****(*****GSE9331*****):** Up-regulated in 21 samples (out of 50 samples).***Phosphate depletion*****(*****GSE14840*****):** Up-regulated in 6 samples (out of 6 samples)
**FACOAL161** (fatty acid CoA ligase hexadecenoate) (*Rv3826* or *Rv1529*)	Fatty Acid Metabolism (FAS161)		
**FACOAL180** (fatty acid CoA ligase octadecanoate) (*Rv3826* or *Rv1529*)	Fatty Acid Metabolism (FAS181)		
**ADA** (Adenosine deaminase) (*Rv3313c*)	Purine Metabolism (PUNP5)		
**HXPRT** (hypoxanthine phosphoribosyltransferase Hypoxanthine) (*Rv3624c*)	Purine Metabolism (PUNP5)		
**ORNt** (ornithine transport) (*Rv2320c*)	Sugar Metabolism (**GMAND**)		
**ME1** (malic enzyme NAD) (*Rv2332*)	Glycolysis **(GAPD, PGK, PYK**)		
**DHNPA** (dihydroneopterin aldolase) (*Rv3607c*)	Folate Metabolism (DHPS2)		***Hypoxia*****(*****GSE9331*****):** Up-regulated in 12 samples (out of 50 samples).
**DESAT16** (Palmitoyl CoA desaturase n C160CoA n C161CoA) (*Rv3229c*)	Fatty Acid Metabolism (FAS161)		***Phosphate depletion*** **(*****GSE14840*****):** Up-regulated in 4 samples (out of 6 samples)

^*^Reactions whose genes showed negative co-expression are marked in underlined bold letters.

**Table 3:** Microarray samples used for gene expression correlations of metabolic genes.

GSE number	Experimental conditions	Number of sample used	Reference
GSE3201	Exponential growth phase	38	Gao *et al.*^53^
GSE6209	Human macrophage infection	11	Fontan *et al.*^57^
GSE8786	Stationary phase and low oxygen dormancy	54	Voskuil *et al.*^54^
GSE8827	Macrophage intracellular cue	8	Rohde *et al.*^56^
GSE9331	Hypoxic condition	52	Rustad *et al.*^32^
GSE10391	Multiple stress conditions	75	Deb *et al.*^33^
GSE11095	Carbon monoxide treatment	6	Shiloh *et al.*^55^
GSE11096	dosR and dosS mutants CO sensing	34	Shiloh *et al.*^55^
GSE14005	With lung surfactant	8	Schwab *et al.*^59^
GSE14840	Phosphate depletion	6	Rifat *et al.*^34^
GSE16146	Reactive oxygen and nitrogen	80	Voskuil and Visconti, 2009 (un-published)
GSE21114	*In vitro* and inside macrophage	116	Homolka *et al.*^58^
GSE21590	Reaeration timecourse from a defined hypoxia model	33	Sherrid *et al.*^60^
