# Corrigendum: Integration of Metabolic Modeling with Gene Co-expression Reveals Transcriptionally Programmed Reactions Explaining Robustness in *Mycobacterium tuberculosis*

**DOI:** 10.1038/srep24916

**Published:** 2016-04-29

**Authors:** Bhanwar Lal Puniya, Deepika Kulshreshtha, Inna Mittal, Ahmed Mobeen, Srinivasan Ramachandran

Scientific Reports 6: Article number: 2344010.1038/srep23440; Published online: 03222016; Updated: 04292016

The Acknowledgements section in this Article was omitted. The Acknowledgements should read:

“We thank Dr. Kausik Chakraborty for thought provoking discussion. BSC0121 is duly acknowledged for providing support for this work. BLP acknowledges CSIR, India for providing fellowship.”

