# Supplementary material for: Integration of Metabolic Modeling with Gene Co-expression Reveals Transcriptionally Programmed Reactions Explaining Robustness in Mycobacterium tuberculosis
Source: Sci Rep. 2016 Mar 22;6:23440. doi: 10.1038/srep23440 (PMC4802306; doi:10.1038/srep23440)
Supplement: Supplementary Information [file srep23440-s1.pdf]

Title: Integration of Metabolic Modeling with Gene Co-expression Reveals Transcriptionally Programmed Reactions Explaining Robustness in *Mycobacterium tuberculosis*

Authors: Bhanwar Lal Puniya, Deepika Kulshreshtha, Inna Mittal, Ahmed Mobeen, and Srinivasan Ramachandran*

Fig S1

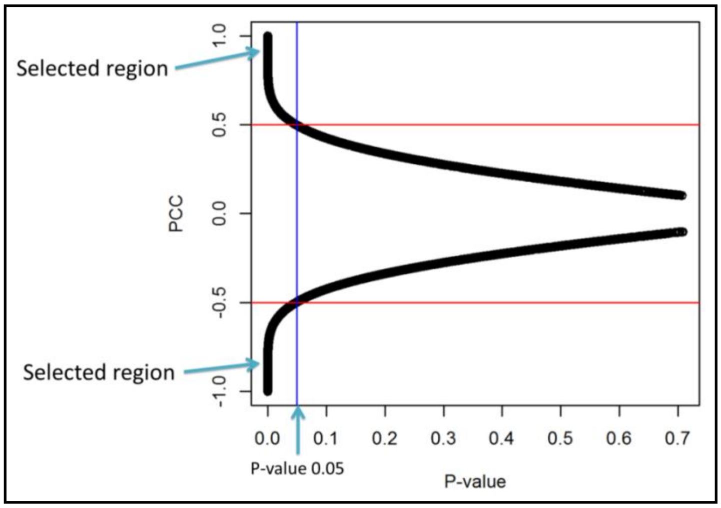

Fig S1 A. The PCC and p-value of correlated reaction sets. PCC above 0.5 and lower than 0.5, all are significant with p-value 0.05 (Selected regions). Red line (Horizontal) is at PCC 0.5 and -0.5, and blue line (vertical) at P-value 0.05.

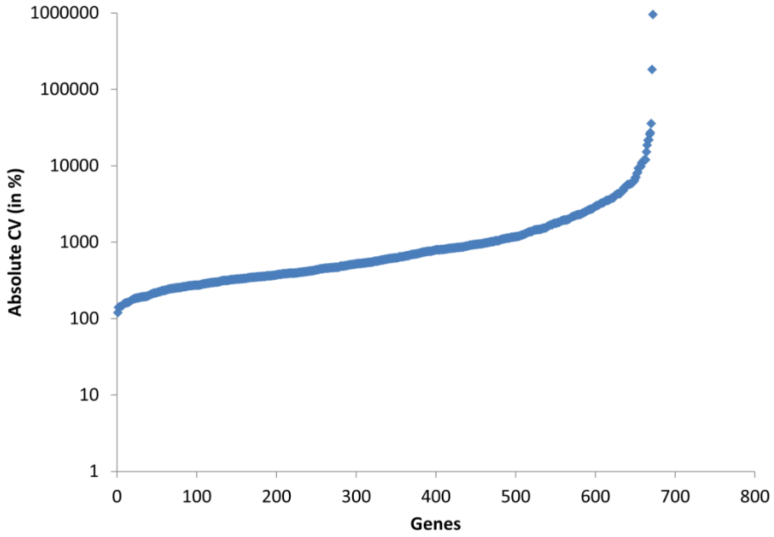

Fig S1 B: Coefficient of variation (CV) of expression values (Z-score) of 672 genes used for network construction. All 672 genes have high CV (above 100%). Plot is shown in semi logarithmic (log [base=10] Y-axis) scale.

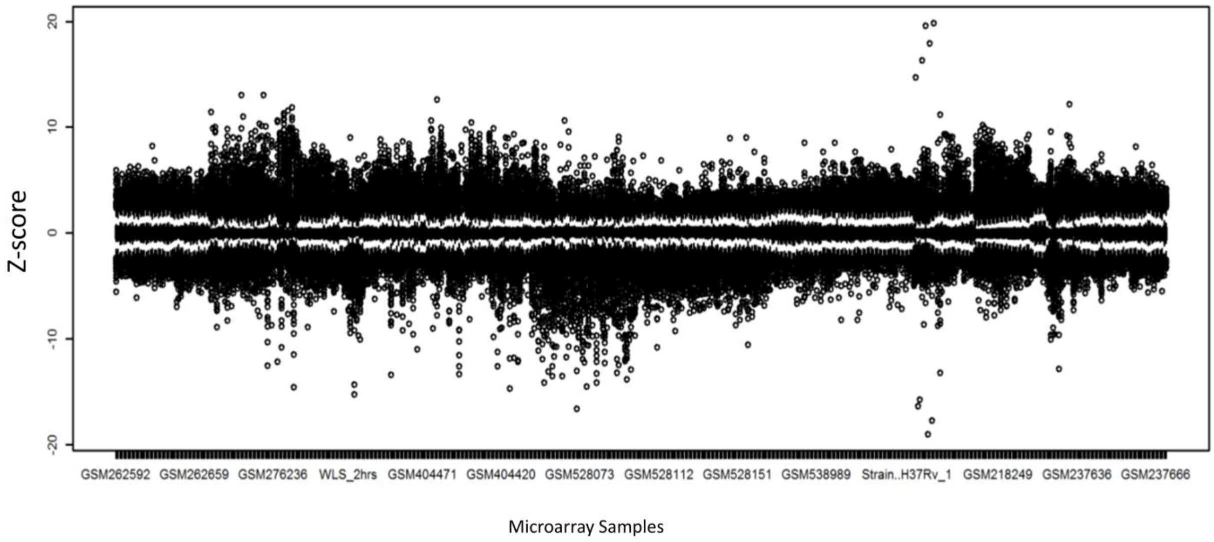

Fig S1 C: Combined boxplot of all 521 samples of 13 different studies after Z-score (mean centered) normalization. X-axis: Microarray Samples, Y-axis: Z-score.

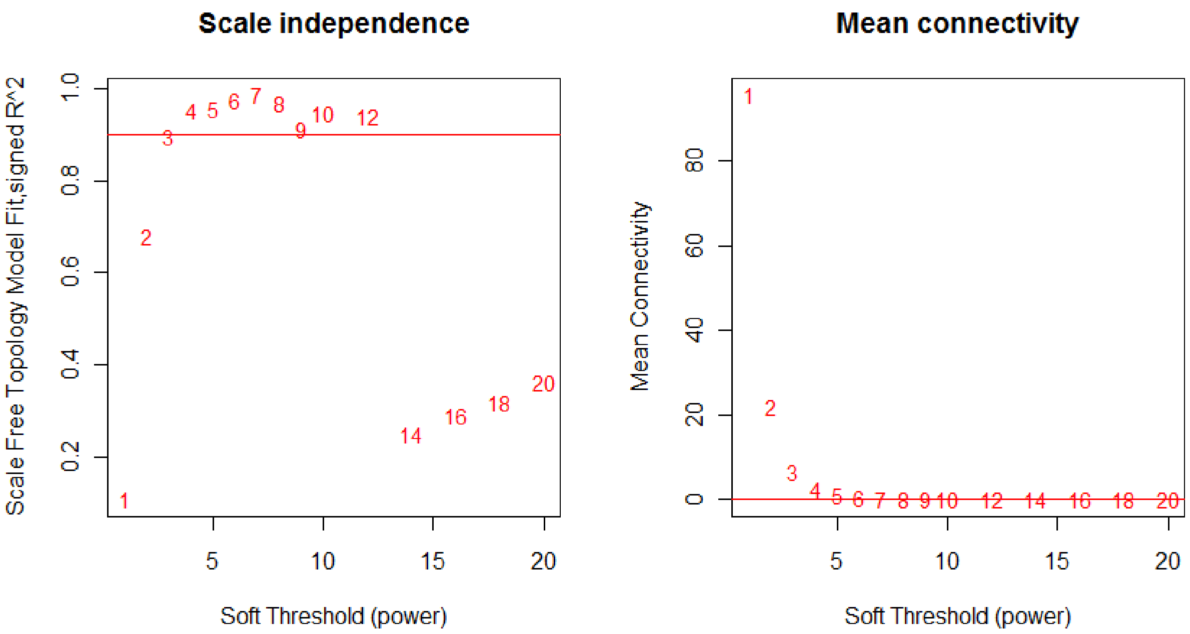

Fig S1 D: Estimating soft thresholding power of gene co-expression network. Horizontal abline is at $R^2 = 0.85$ in right panel and at 0 in left figure.

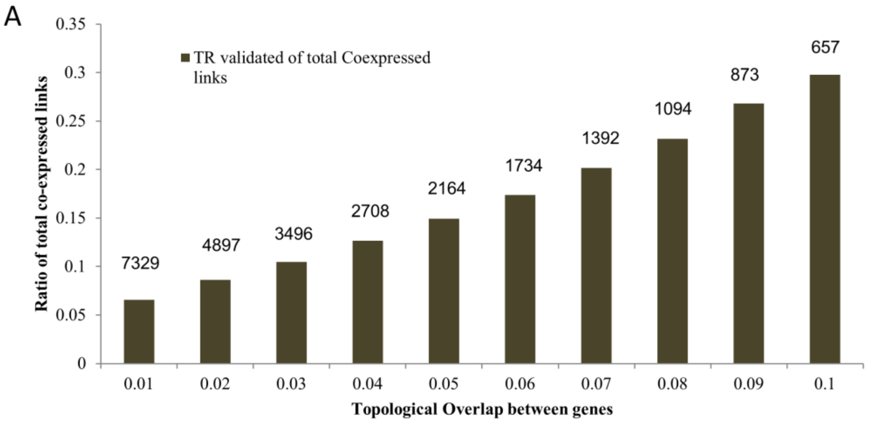

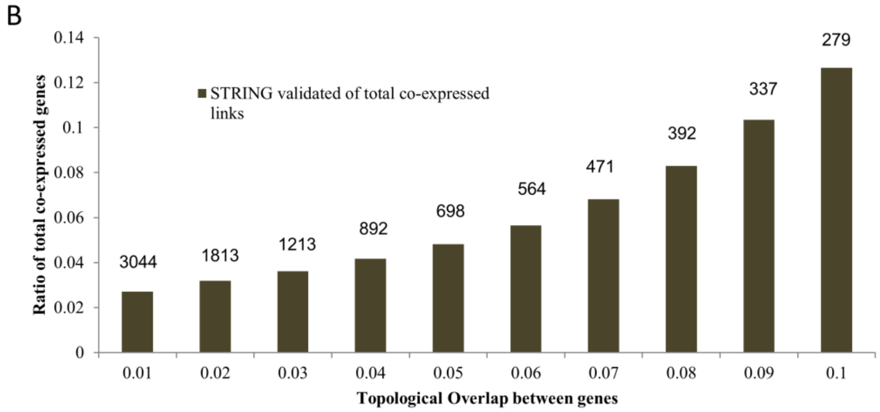

Fig S1 E: Distribution of supported co-expression links with (A) Transcriptional Regulatory Network and (B) STRING database. The data show that number of supported connections with both datasets (written above bars) were decreasing with increased topological overlap of the connections between genes, whereas the ratio of supported connections (height of the bars) out of total co-expressed connections increases with increasing topological overlap between genes. Connections with higher TO values between genes are better supported through complementary evidences or data.

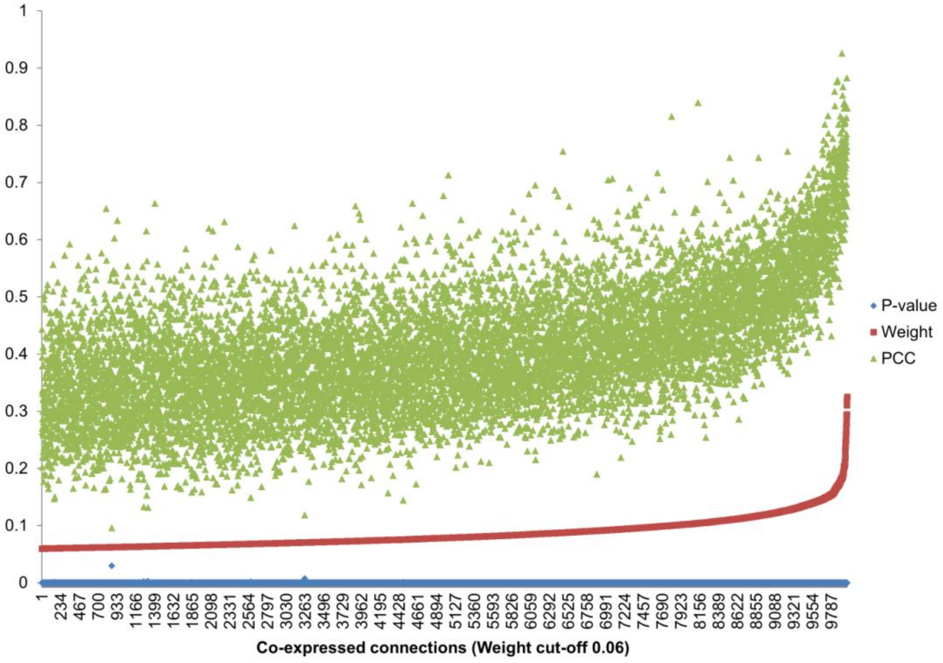

Fig S1 F: P-value and PCC of connections of TO weight threshold 0.06. All connections above weight threshold of 0.06 have significant PCC.
